# Serum SDF-1 levels are a reliable diagnostic marker of feline mammary carcinoma, discriminating HER2-overexpressing tumors from other subtypes

**DOI:** 10.18632/oncotarget.22398

**Published:** 2017-11-11

**Authors:** Cláudia S. Marques, Maria Soares, Ana Santos, Jorge Correia, Fernando Ferreira

**Affiliations:** ^1^ Center for Interdisciplinary Research in Animal Health, Faculty of Veterinary Medicine, University of Lisbon, 1300-477 Lisbon, Portugal

**Keywords:** feline mammary carcinoma, SDF-1, serum biomarker, HER2, CXCR4

## Abstract

The feline mammary carcinoma (FMC) is the third most common tumor in cat, sharing many clinicopathological features with human breast cancer and thus, considered a suitable model for comparative oncology. Due to its poor prognosis, further studies are required to improve the diagnostic accuracy and treatment of cats with spontaneous mammary carcinoma. Recently, it was reported that the overexpression of stromal cell-derived factor-1 (SDF-1) has great value in human breast cancer diagnosis, suggesting that diagnostic tools and therapies targeting the SDF-1 ligand can improve the clinical outcome. In this study, we aimed to evaluate if serum SDF-1 levels can also be used as a biomarker of mammary carcinoma in cats and to analyze if serum SDF-1 levels are associated with clinicopathological features, linked to a specific FMC subtype or correlated with the tumor expression of SDF-1 receptor, the chemokine C-X-C motif receptor 4 (CXCR4). Results showed that cats with mammary carcinoma had significantly higher serum SDF-1 levels than healthy controls (*p*=0.035) and ROC analysis revealed that the best cut-off value to differentiate sick from healthy animals was 2 ng/ml (specificity: 80%; sensitivity: 57%; AUC=0.715). Significant associations were also found between cats with elevated serum SDF-1 concentrations (≥ 2 ng/ml) and HER2-overexpressing mammary carcinomas (Luminal B-like and HER2-positive subtypes, *p*<0.0001), CXCR4-negative mammary carcinomas (*p*=0.027), mammary carcinomas with small size (<3 cm, *p*=0.027) and tumors with low Ki-67 expression (*p*=0.012). No statistical associations were found between serum SDF-1 levels and overall or disease-free survival. In summary, our results show that serum SDF-1 levels can be used as a biomarker of feline mammary carcinoma, especially in cats with HER2-overexpressing mammary tumors. Data suggest that targeted therapies against the SDF-1 ligand and/or its CXC4 receptor may be effective for the treatment of FMC, as described for human breast cancer, strengthening the concept that spontaneous feline mammary carcinoma is a suitable model for comparative oncology.

## INTRODUCTION

The feline mammary carcinoma (FMC) is very common in cat (12-40% of all neoplasms), showing overexpression of the HER2 protooncogene in 33%-60% of the cases [1–5]. Sharing phenotypic and genotypic similarities with human breast tumor [6–10], spontaneous FMCs can also be classified in the same subtypes (luminal A, luminal B, luminal B-like, epidermal growth factor receptor type II-positive and triple negative) [7, 11], being considered a suitable model for breast cancer studies.

Despite the efforts to understand the oncogenic mechanisms of breast cancer, the discovery of more accurate biomarkers and therapeutic targets are listed as research priorities [12]. Considering this scenario, an increasing attention is given to a family of chemotactic molecules known as chemokines, which are secreted by a variety of stromal and epithelial cells, exerting its biological effects by interacting with G protein-coupled receptors (GPCR). Their binding to these receptors mediates leukocyte migration and adhesion to the tumor endothelial cells, regulating the tumor growth, angiogenesis and apoptosis [13, 14]. Indeed, recent studies showed that the binding of stromal cell-derived-factor-1 (SDF-1), also known as chemokine ligand 12 (CXCL12), to the C-X-C chemokine receptor 4 (CXCR4) increases the proliferation rates and stromal vascular endothelial growth of several types of cancer [15–17], including breast tumors [18–23]. Moreover, since the SDF-1/CXCR4 axis elicits the activation of multiple kinase pathways (e.g. PI3K, MAPK, ERK1/2), its inhibition may represent a new therapeutic strategy to treat mammary tumors more effectively. Besides, the CXCR4 overexpression has been observed in metastatic breast cancer patients [24–28], especially in HER2-overexpressing [29–33] and triple negative breast tumors [34–39], suggesting that SDF-1 receptor inhibition might constitute a novel therapeutic approach. Regarding the expression of the SDF-1, little is known. Nevertheless, some studies reported that breast cancer patients display increased serum SDF-1 levels, in particular patients with HER2-overexpressing breast tumors [40] and that serum SDF-1 concentration has prognostic value [19, 40–44].

In cat, only scarce data is available on the molecular mechanisms underlying the SDF-1/CXCR4 axis. In 2002, Tanabe at al. [45] showed that CXCR4 is overexpressed in 72% cases of feline mammary adenocarcinomas and 10 years later, Ferrari and et al. [46] demonstrated that SDF-1/CXCR4 axis has a proliferative role in feline mammary carcinoma cells. Taking into account the oncogenic role of the SDF-1/CXCR4 axis in human breast cancer and the promising data on its inhibition, we aimed to determine if the serum SDF-1 levels have diagnostic value in cats with mammary carcinoma and to calculate the best cut-off value, which allows to discriminate between sick and healthy animals. Finally, the statistical associations between serum SDF-1 levels and different FMC subtypes, tumor's CXCR4 expression and clinicopathological features were estimated, in order to better understand the clinical relevance of the SDF-1 ligand in cat, towards the development of diagnostic tools and targeted therapies.

## RESULTS

### Animal study population

The main clinicopathological features of the cats with mammary carcinoma enrolled in the study (n=42), are summarized in Table 1. The mean age at diagnosis was 11.51 ± 2.62 years ranging from 7 to 16.5 years. All animals were submitted to surgical mastectomy and four (10%) were subjected to anthracycline-based adjuvant chemotherapy (doxorubicin, 25 mg/m2, intravenously, every 3 weeks for 5 cycles). Eleven queens (26%) showed HER2-overexpressing mammary carcinomas, whereas fourteen cats (33%) had elevated serum HER2 levels (Table 1). The overall survival (OS) was 16.39 ± 10.16 months (n=41) and the survival ratio was 50%. The disease free-survival (DFS) was 12.15 ± 7.84 months (n=34) and nineteen (56%) of the cats with mammary carcinoma had disease recurrence at the end of the follow-up period (54 months), with 50% of the cats showing locoregional recurrence (n=17) and 6% displaying distant metastases (n=2).

**Table 1:** Clinicopathological features of the cats with mammary carcinoma enrolled in the study

Clinicopathological feature	No. of animals (%)	Clinicopathological feature	No. of animals (%)
**Breed**		**Tumor Size**	
Not determined	30 (71%)	≤ 3 cm	31 (74%)
Siamese	7 (17%)	>3 cm	11 (26%)
Persian	3 (7%)	**HP^a^ classification**	
Norwegian Forest Cat	2 (5%)	Papillary-cystic carcinoma	2 (5%)
**Spayed**		Cribriform carcinoma	3 (7%)
No	25 (60%)	Mucinous carcinoma	5 (12%)
Yes	16 (38%)	Solid carcinoma	7 (16%)
Unknown	1 (2%)	Tubular carcinoma	10 (24%)
**Contraceptives**		Tubulopapillary carcinoma	15 (36%)
No	13 (31%)	**Malignancy grade**	
Yes	21 (50%)	I	2 (5%)
Unknown	8 (19%)	II	10 (24%)
**Treatment**		III	30 (71%)
Mastectomy	38 (90%)	**Necrosis**	
Mastectomy + Chemo	4 (10%)	No	17 (40%)
**Multiple tumors**		Yes	25 (60%)
No	12 (29%)	**Lymphatic invasion**	
Yes	30 (71%)	No	35 (83%)
**Lymph node status**		Yes	7 (17%)
Negative	23 (55%)	**Lymphocytic infiltration**	
Positive	14 (33%)	No	20 (48%)
Unknown	5 (12%)	Yes	22 (52%)
**Tumor stage (TNM)**		**Tumor ulceration**	
I	11 (26%)	No	40 (95%)
II	7 (17%)	Yes	2 (5%)
III	22 (52%)	**Ki 67 index**	
IV	2 (5%)	Low (< 14%)	15 (36%)
**Localization**		High (≥ 14%)	26 (62%)
M1	9 (22%)	Unknown	1 (2%)
M2	9 (22%)	**PR status**	
M3	17 (40%)	Negative	22 (52%)
M4	6 (14%)	Positive	20 (48%)
Unknown	1 (2%)	**ER status**	
**Recurrence**		Negative	25 (60%)
No	14 (33%)	Positive	17 (40%)
Yes	20 (48%)	**HER2 status**	
Unknown	8 (19%)	Negative	31 (74%)
**Survival (42 months follow-up)**		Positive	11 (26%)
No	21 (50%)	**Serum HER2 levels**	
Yes	20 (48%)	Negative (< 10 ng/ml)	21 (50%)
Unknown	1 (2%)	Positive (≥ 10 ng/ml)	14 (33%)
		Unknown	7 (17%)

^a^ Histopathological.

### Cats with mammary carcinoma showed higher serum SDF-1 levels than healthy cats

**Figure 1:**
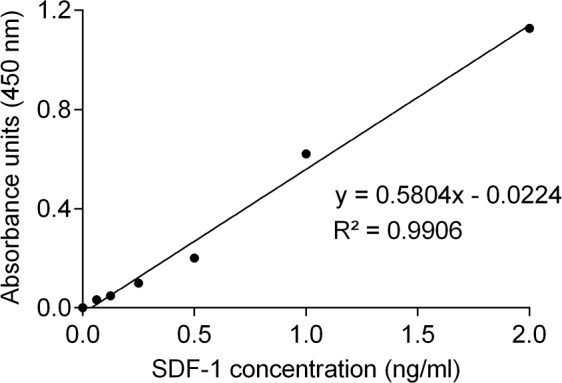
Standard curve for quantification of serum SDF-1 levels by ELISA Concentrations of SDF-1 were calculated by replacing the X in the trend line equation by the average of the absorbance units measured in duplicate for each sample. R-square (R^2^) value of the linear regression was higher than 0.99.

**Figure 2:**
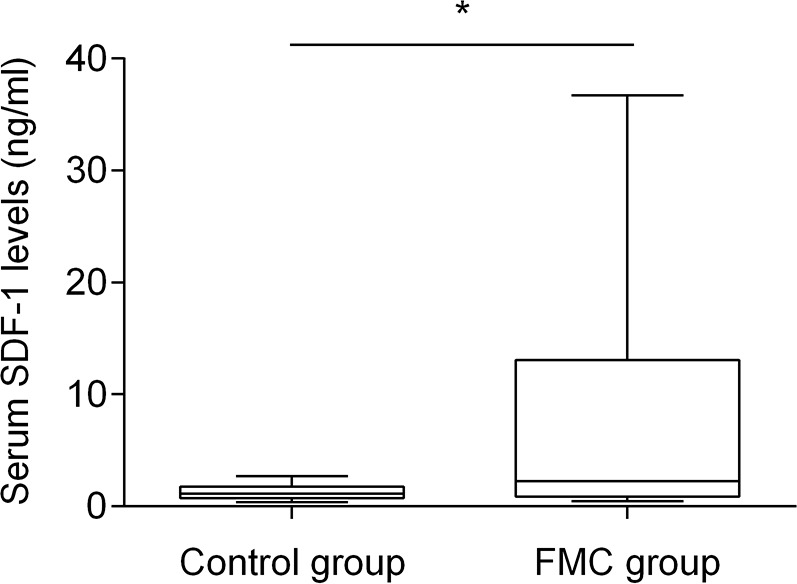
Box plot diagrams showing that queens with mammary carcinoma (FMC group) have higher serum SDF-1 levels than healthy cats (control group) The non-parametric Mann–Whitney U test was used to compare serum SDF-1 levels between the two groups. ^*^ indicates significant difference (*p*=0.035).

Knowing that human SDF-1 and feline SDF-1 share 96% amino acid identity, serum SDF-1 levels were measured using a commercial ELISA kit and following the manufacturer's recommendations. Serum SDF-1 concentrations were then calculated from the standard curve generated by serial dilutions of recombinant SDF-1 protein with known concentrations (Figure 1, R^2^=0.99). Results revealed that cats with mammary carcinoma exhibited higher serum SDF-1 levels (mean value of 8.76 ng/ml; range of values: 0.45-36.72 ng/ml) than healthy cats (mean = 1.28 ng/ml; range of values: 0.38-2.69) ng/ml), with a significant *p*-value of 0.035 (Figure 2).

### Serum SDF-1 levels ≥ 2 ng/ml give the best cut-off value to diagnose cats with mammary carcinoma

**Figure 3:**
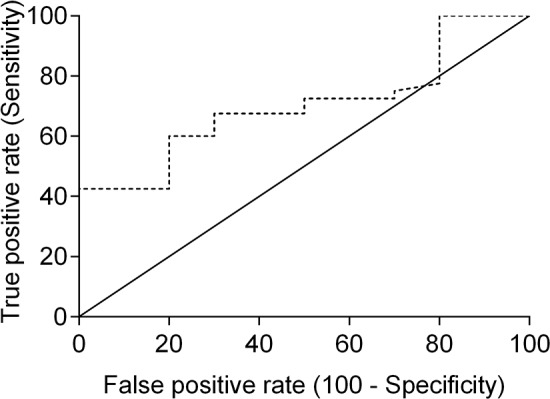
Receiver-operating characteristic (ROC) curve of serum SDF-1 levels for ELISA The best SDF-1 cut-off value (2 ng/ml) was determined to maximize the sum of the sensitivity (57%) and specificity (80%) using the Youden index (sensitivity + specificity - 1). The estimated AUC was 0.715 ± 0.076 (95% CI: 0.566-0.865, *p*=0.036).

Receiver-operating characteristics (ROC) analysis was performed to determine the best cut-off point for serum SDF-1 levels, using ELISA as a diagnostic tool. ROC analysis revealed that 2 ng/ml is the best cut-off value to discriminate cats with mammary carcinoma from healthy ones (Figure 3), with a specificity of 80%, a sensitivity of 57% and an area under the curve (AUC) of 0.715 (95% CI: 0.566-0.865, SEM=0.076). To reinforce the utility of measuring serum SDF-1 levels in cats with mammary carcinoma, a nonparametric method that does not make any assumptions about the distribution of ELISA results in both groups was used and a significant *p-*value was obtained (*p*=0.036). Indeed, only two of the ten healthy cats (20%) showed serum SDF-1 levels ≥ 2 ng/ml, whereas the majority of cats with mammary carcinoma exhibited elevated serum SDF-1 levels (Table 2).

**Table 2:** Serum SDF-1 levels in healthy cats and in cats with mammary carcinoma, measured by ELISA

	Animals (%)	Mean and range values (ng/ml)
**Healthy cats (n=10)**		
Low levels (< 2ng/ml)	8 (80%)	0.93 (0.38-1.44)
High levels (≥ 2 ng/ml)	2 (20%)	2.69 (2.58-2.80)
**Sick cats (n=42)**		
Low levels (< 2 ng/ml)	17 (40%)	0.84 (0.45-1.60)
High levels (≥ 2 ng/ml)	25 (60%)	14.14 (2.00-36.72)

### Elevated serum SDF-1 levels are associated with HER2-overexpressing feline mammary carcinomas

**Figure 4:**
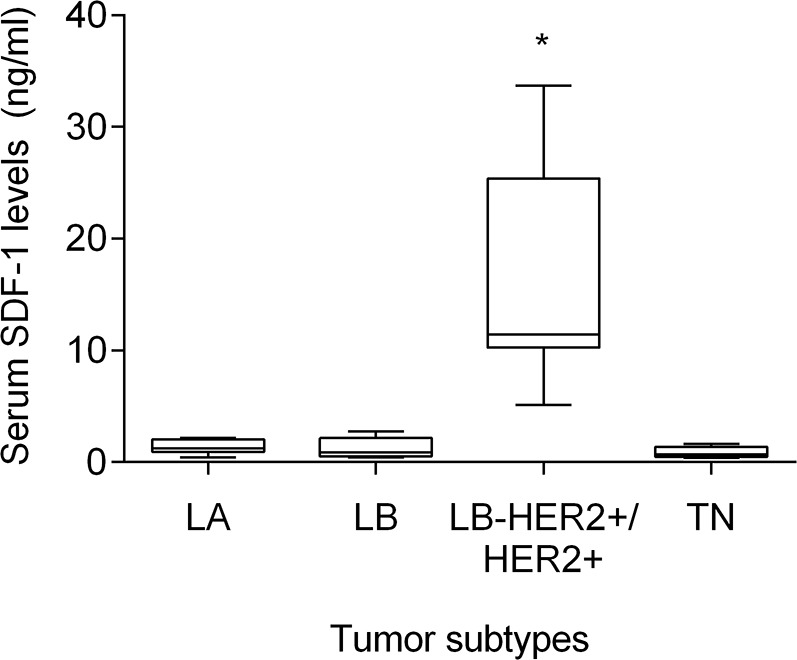
Cats with HER2-overexpressing mammary carcinoma show higher serum SDF-1 levels Mammary carcinomas were classified accordingly to the St. Gallen International Expert Consensus panel guidelines. To compare circulating SDF-1 levels between animals with different FMC subtypes (*LA -* luminal A; *LB* - luminal B; *LB-HER2+/HER2+* - luminal B-like/HER2-positive; *TN*-normal and basal triple-negative), the non-parametric Kruskall-Wallis test and Dunn's multiple comparisons post-test were used. ^*^ indicates significant differences between cats with HER2-overexpressing mammary carcinomas (*LB-HER2+/HER2+*) and cats with other FMC subtypes (*p*<0.05).

**Figure 5:**
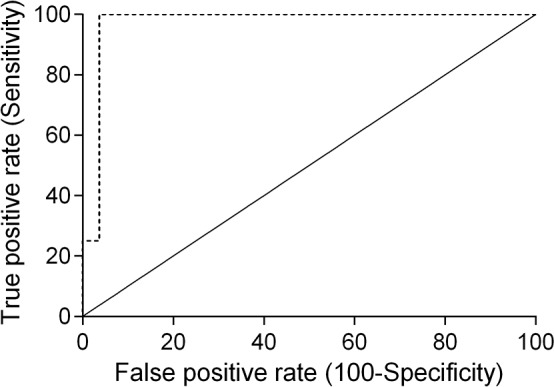
Receiver-operating characteristic (ROC) curve of serum SDF-1 levels of cats with HER-2-overexpressing and HER-2 negative tumors The best cut-off value (4 ng/ml) was determined to maximize the sum of the sensitivity (100%) and specificity (96%) using the Youden index (sensitivity + specificity - 1). The estimated AUC was 0.972 ± 0.028 (95% CI: 0.917-1.027, *p*<0.0001).

**Figure 6:**
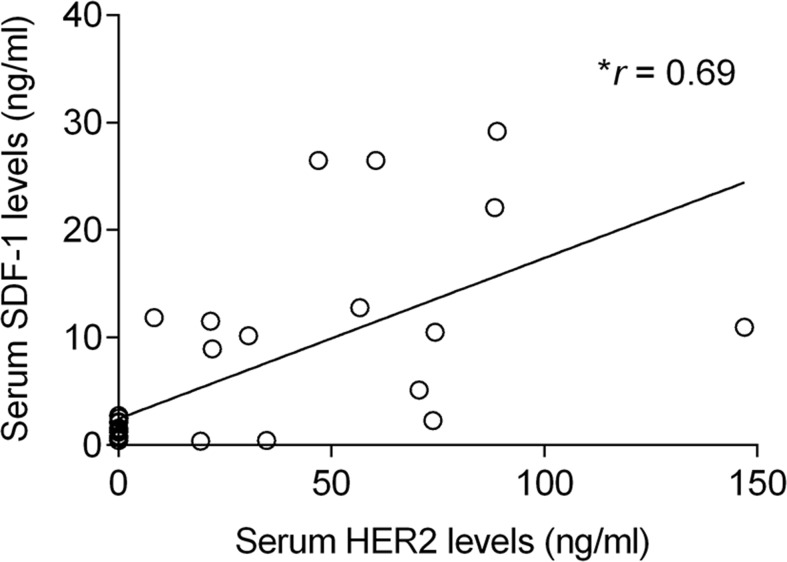
Elevated serum SDF-1 levels are associated with high circulating HER2 levels in cats with mammary carcinoma The non-parametric Spearman rank test was used to assess the correlation between the serum SDF-1 levels and serum HER2 levels. *r* means the correlation coefficient value and ^*^ indicates significant correlation between the two variables (*p*<0.0001).

Cats enrolled in this study were grouped in four clusters based on their tumor subtype, in order to determine if serum SDF-1 levels are correlated with a specify mammary carcinoma immunophenotype. Cats with HER2-overexpressing mammary carcinomas (Luminal B-like and HER2-positive subtypes) showed higher serum SDF-1 levels (16.07 ± 9.26 ng/ml) than cats with mammary carcinoma from other subtypes (*p*<0.001, Figure 4). Further ROC analysis revealed that 4 ng/ml is the best cut-off value to differentiate cats with HER2-overexpressing phenotype and cats presenting other molecular subtypes (Figure 5), with a specificity of 96%, a sensitivity of 100% and a significant area under the curve (AUC) of 0.972 (95% CI: 0.917-1.027, SEM=0.028, *p*<0.0001). In addition, a positive correlation was found between serum SDF-1 levels and serum HER2 levels (*r*=0.69, 95% CI: 0.46 - 0.84, *p*<0.0001, Figure 6), corroborating the previous results, since the serum HER2 levels are associated with tumor HER2 status [11].

### Cats with CXCR4-overexpresing mammary carcinomas showed low serum SDF-1 levels

**Figure 7:**
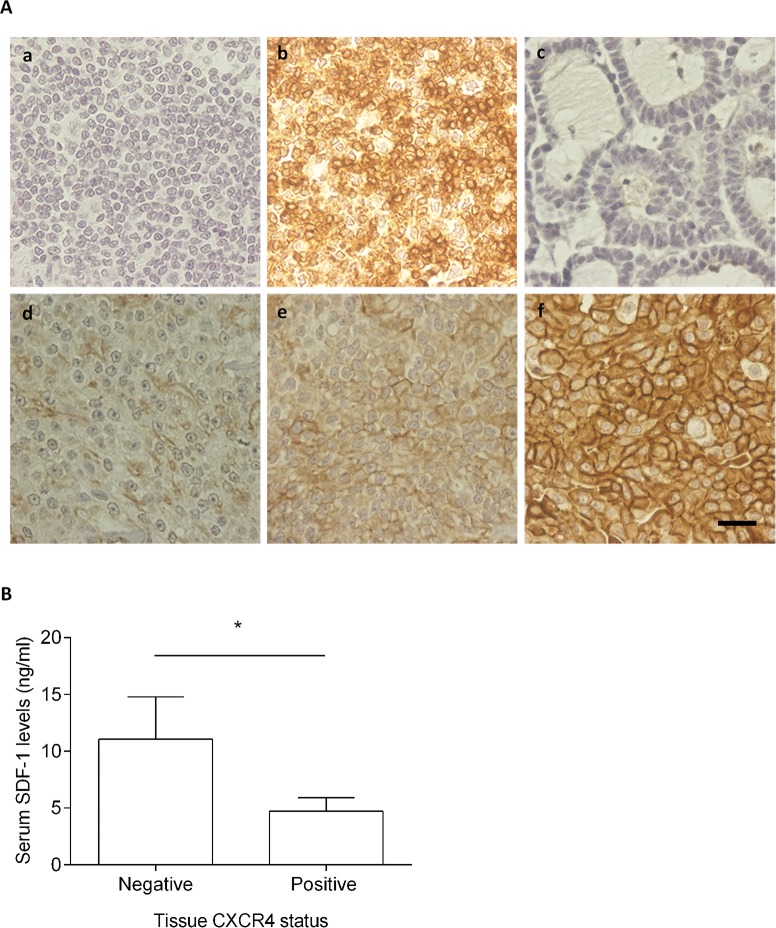
Cats with CXCR4-positive mammary carcinoma show low serum SDF-1 levels **(A)** The CXCR4 expression was assessed by immunohistochemistry using normal feline lymphoid tissue (tonsil) as negative (a) and positive controls (b). Healthy mammary tissue scored as 0 (c) and samples of mammary carcinomas scored as 1+, 2+ and 3+ (d-f) are presented. The total magnification is 400x and the scale bar represents 20μm. **(B)** The non-parametric Mann-Whitney was used to compare serum SDF-1 levels between the cats having CXCR4-negative mammary carcinoma and cats with CXCR4-overexpressing mammary carcinoma. Bars represent the mean value ± SEM and ^*^ indicates a significant difference (*p*=0.027).

The CXCR4 expression was evaluated by using a semi-quantitative scoring system previously published [19, 21, 26, 46]. Briefly, the staining intensity (weak, moderate, strong) and the number of labeled tumor cells were analyzed (Figure 7A), with the CXCR4-negative tumor samples being scored as 0 (Figure 7A, c) or 1+ (Figure 7A, d), and CXCR4-positive tumor samples classified as 2+ (Figure 7A, e) or 3+ (Figure 7A, f). From the 42 feline mammary carcinomas evaluated, 6 (14%) samples were classified as CXCR4-negative samples and 36 (86%) as CXCR4-positive samples, with CXCR4-negative mammary carcinoma showing significant higher serum SDF-1 levels (*p*=0.027, Figure 7B).

### Cats with elevated serum SDF-1 levels showed less aggressive clinicopathological features

Significant associations were found between cats with elevated serum SDF-1 levels (≥ 2 ng/ml) smaller mammary carcinomas (≤ 3 cm; *p*=0.012; OR = 0.068; 95% CI: 0.007-0.63) and tumors with lower Ki-67 index (< 14%, *p*=0.037; OR = 0.185; 95% CI: 0.04-0.84) (Table 3). In addition, cats with elevated serum SDF-1 levels (≥ 2 ng/ml) were associated with cats showing HER2-positive mammary carcinomas (*p*=0.0001; OR = 53.67; 95% CI: 2.78 - 1034). Finally, no significant differences in overall and disease-free survival rates were found between cats with mammary carcinoma showing increased serum SDF-1 levels and cats with mammary carcinomas exhibiting decreased serum SDF-1 levels.

**Table 3:** Associations between serum SDF-1 levels and clinical pathological features

	Serum SDF-1 levels		
	Low (< 2 ng/ml) (n)	High (≥ 2 ng/ml) (n)	*p* Values and odd ratios (OR)
**Size class**			
≤ 3 cm > 3 cm	10 7	21 1	*p* = 0.012 OR = 0.068
**Recurrence**			
No Yes	11 6	12 5	*p* = 1.000
**Alive**			
No Yes	6 11	12 5	*p* = 1.000
**Multitumors**			
No Yes	14 3	19 3	*p* = 0.078
**Necrosis**			
No Yes	5 11	12 10	*p* = 0.197
**Lymphatic vessel invasion**			
Yes No	3 14	3 19	*p* = 1.000
**Lymphocytic infiltration**			
No Yes	9 8	11 13	*p =* 0.756
**Lymph node metastasis**			
No Yes	11 6	12 5	p = 1.000
**Ki-67 index**			
Low (< 14%) High (≥ 14%)	4 13	10 6	*p* = 0.037 OR =0.185
**PR status**			
Negative Positive	9 8	13 10	*p* = 1.000
**ER status**			
Negative Positive	11 6	10 11	*p* = 0.341
**HER2 status**			
Negative (scores 0 and 1+) Positive (scores 2+ and 3+)	17 0	7 11	*p* = 0.0001 OR = 53.67

## DISCUSSION

Due to the similarities between human breast cancer and feline mammary carcinoma [6–10], new clinical studies on FMC may identify novel diagnostic markers and therapeutic targets that can probably be used in human patients. Recently, it was demonstrated that stroma cells of breast tumors synthesize the chemokine SDF-1, which via its cognate receptor (CXCR4) supports tumor growth through autocrine and paracrine mechanisms [14, 19, 24, 41, 47]. Moreover, it was showed that breast cancer cells overexpressing CXCR4 metastasize to distant sites, where SDF-1 is highly expressed [18, 48], raising the possibility that targeted therapies against SDF-1/CXCR4 axis may inhibit tumor growth, as described *in vitro* [49–54]. However, despite these promising findings, the value of serum SDF-1 levels in the diagnosis and their correlation with clinicopathological features are unknown in cats and poorly understood in humans.

In the present study, we demonstrated that serum SDF-1 levels have diagnostic value in cats with mammary carcinoma and determined that the best cut-off value to discriminate cats with mammary carcinoma from healthy ones (≥ 2 ng/ml) is close to the one reported for breast cancer patients [40, 43], reinforcing the idea that FMC is a suitable cancer model. Furthermore, a significant association was found between cats with HER2-overexpressing mammary carcinomas (luminal B-like and HER2+ subtypes) and cats with high serum SDF-1 levels, as reported for breast cancer patients [40, 43]. Indeed, cats with elevated serum SDF-1 levels have 53 times more likelihood to show HER2-positive tumor status and increased serum HER2 values (*p*<0.0001), corroborating the recent data that associates the HER2 tumor status with the serum HER2 levels [11], as also reported in humans [55, 56]. Further ROC analysis also revealed a cut-off value of 4 ng/ml in serum levels, that allows differentiation of HER-2 positive from HER-2 negative tumor samples. Considering the proliferative role of SDF-1/CXCR4 axis in breast tumors [27, 28] and the promising *in vitro* results obtained with CXCR4 inhibitors, the expression of the SDF-1 receptor was also evaluated in feline mammary carcinomas. The immunohistochemical analysis revealed that CXCR4 is overexpressed in the majority of FMC (36/42; 86%), as previously reported in cat [46] and in breast cancer patients [27, 28]. In addition, cats with CXCR4-positive mammary carcinomas showed lower serum SDF-1 levels then cats with CXCR4-negative mammary carcinomas (*p*=0.027), uncovering a putative negative feedback of the SDF-1 ligand on SDF-1/CXCR4 axis. In breast cancer patients it was reported that low serum SDF-1 levels may favor the migration of tumor cells overexpressing CXCR4, promoting the development of distant metastasis [19]. Interestingly, we found that cats with elevated serum SDF-1 levels are associated with mammary carcinomas showing smaller size (≤3 cm; *p*=0.012), lower ki-67 index (< 14%; *p*=0.037) and HER2 overexpression (*p*=0.0001), with 9 from 11 HER2-overexpressing mammary carcinomas (81.8%), being classified as luminal B-like subtype, which is associated with a better outcome than HER2 subtype, both in cat [7] and humans [57–60].

So far, few attempts were made to correlate the expression levels of the SDF-1 receptor, CXCR4 and the different molecular subtypes in human breast cancer. Nevertheless, an elegant study established a functional link between the HER2 and CXCR4 signaling pathways, being the PI3K/Akt/mTOR pathway activation responsible for the HER2-induced CXCR4 expression and the HER2 implied in the inhibition of the SDF-1-induced CXCR4 ubiquitination [32]. Very recently, it was also found that CXCR4 could be a very promising therapeutic target in patients with HER2-overexpressing breast cancer patients, since CXCR4 inhibitors efficiently reduced tumor growth and metastasis in both Herceptin-sensitive and Herceptin-resistant HER2 patient-derived xenografts [33]. Further studies on SDF-1 and CXCR4 gene expression in primary and metastatic HER-2 tumors are in need to be performed in order to gain a better knowledge on how SDF-1/CXCR4 axis is acting at the molecular level in woman and female cat.

In summary, our work identified a new serum biomarker for feline mammary carcinoma, in particular for HER2-tumors, opening new perspectives for the development of diagnostic tools and design of new therapies targeting the SDF-1/CXCR4 axis. Further, the results also reinforce the scenario that FMC is a suitable spontaneous cancer model which may allow to predict novel therapeutic strategies in humans.

## MATERIALS AND METHODS

### Sample collection

Forty-two blood samples from cats with spontaneous mammary carcinomas and 10 from healthy cats, that underwent surgical treatment at the Small Animal Hospital of the Veterinary Medicine Faculty, University of Lisbon (FVM-ULisboa), were selected in a prospective study from June 2012 to December 2016, after the owner's cat permission.

For each animal, the following clinicopathological features were recorded: age, breed, reproductive status, progestogens administration, prescribed treatment (none, surgery, surgery plus chemotherapy), number and location of tumor lesions, tumor size, lymph node status, histopathological classification, malignancy grade, presence of tumor necrosis, lymphatic vessel invasion by tumor cells, lymphocytic infiltration, cutaneous ulceration, regional lymph node involvement, stage of the disease (TNM system) [61], disease-free survival (DFS) and overall survival (OS) were recorded.

Serum was separated from clotted blood by centrifugation (1500g, 10 min, 4°C) and immediately frozen at −80°C, until use. All samples that showed hemolysis were discarded, as recommended for humans [62]. Excised mammary glands, mammary tumors and regional lymph nodes from the animals were immediately fixed in 10% formalin neutralized with 0.1 M phosphate buffer (pH 7.2), during a period no longer than 48 hours. All samples were embedded into paraffin blocks and serial histological sections of 3 μm thickness were prepared, prior to hematoxylin and eosin staining. Carcinomas are classified according to the WHO system adapted by Misdorp et al., 1999 [63] and the degree of malignancy was assessed according to the Elston and Ellis grading system [64], which classifies tumors into grade I (well differentiated), grade II (moderately differentiated), and grade III (poorly differentiated).

### Tissue HER2, ER, PR, Ki-67 and CXCR4 status assessment by IHC

A representative area of each FMC with a diameter of 0.6 cm was selected and tissue sections of 3μm thickness were mounted on glass slides (Star Frost adhesive glass slides, Thermo Scientific, Rockford, USA), placed for 1h at 65°C and overnight at 37°C to properly bind the tissue to the glass. Then tissue samples were deparaffinized with xylene and rehydrated through graded alcohols series to distilled water. For HER2, ER and Ki-67 immunostaining, antigen retrieval was performed by immersing glass tissue slides in citrate buffer (0.01M NaCH3COO, pH 6.0) and using a pressure cooker (2 min at 2 atm), while for PR immunodetection, an immersion in water bath (60 min at 95°C) was performed, as previous reported in [7] and references therein. For CXCR4 detection, tissue slides were immersed in Novocastra^TM^ epitope retrieval solution pH 9 (Leica Biosystems, Wetzlar, Germany) and boiled (25 min at 600W) in a microwave for heat induced epitope retrieval. Then slides were cooled for 30 min at room temperature and rinsed twice for 5 min in PBS. Afterwards, the endogenous peroxidase activity was inhibited by an incubation with hydrogen peroxide (3–4% v/v) during 15 min followed by a protein block (0.4% casein in PBS, with stabilizers, surfactant, and 0.2% Bronidox) for 10 min.

Tissue samples were then incubated at 4°C overnight, in a humidified chamber, with the following primary antibodies: mouse anti-HER2 (clone CB11, 1:200, Invitrogen, Carlsbad, CA, USA), mouse anti-ER (clone 6F11, 1:125, Thermo Scientific), rabbit anti-PR (clone 1E2, ready-to-use, Ventana, Tucson, USA), rabbit anti-Ki-67 (polyclonal, 1:500, Thermo Scientific) and rabbit monoclonal anti-CXCR4 antibody (clone UMB2, Abcam, Cambridge, UK), diluted 1:500 in Lab Vision™ Antibody Diluent OP Quanto (Thermo Fisher Scientific Inc., Waltham, USA). The staining was performed using a modified streptavidin-peroxidase conjugate method based on the poly-HRP anti-rabbit IgG detection system (Novolink™ Polymer Detection System, Leica Biosystems, Wetzlar, Germany), following the manufacturer's guidelines. The peroxidase activity was developed with DAB chromogen (1.74% w/v 3,3 diaminobenzidine) in Novolink™ DAB Substrate Buffer (buffered solution containing ≤0.1% hydrogen peroxide and preservative) for 5 min.. Finally, tissue sections were counterstained for 2 min with Mayer's hematoxylin (Merck, New Jersey, USA), dehydrated and mounted with Entellan® mounting medium (Merck Millipore, Darmstadt, Germany). HER2 immunoreactivity was scored according to the American Society of Clinical Oncology's recommendations. Briefly, FMC were classified as HER2-negative when scored 0 or +1 and HER2-positive if scored as +2 or +3. Mammary carcinomas were also evaluated for ER/PR status using the Allred score system, and only tumors with a score ≥ 2 were considered positive. The Ki-67 proliferation index was determined by dividing the number of tumoral cells showing positive nuclear immunostaining per 1000 tumor cells analyzed over at least three high-amplified microscopic fields. Tumors were considered highly proliferative when more than 14% of the neoplastic cells nuclei expressed Ki-67, as previously reported.

The scoring system for CXCR4 was adopted from previous studies in humans and cats [19, 21, 26, 46]. The intensity of cell membrane and/or cytoplasm staining was graded as 0 (negative), 1 (weak), 2 (moderate) and 3 (strong). The percentage of staining cells obtained, evaluating at least 1000 neoplastic cells in 10 high-power fields (400× magnification) for each tissue section, was also classified as 0 = negative, 1 = <10%, 2 = 10–50%, and 3 = >50%. Multiplication of intensity and percentage scores were used to determine the staining index (0, 1, 2, 3, 4, 6, and 9) and the final results were categorized as reported: staining indexes 0 and 1 were considered CXCR4-negative (0), as the staining indexes 2 and 3 (1+), while the staining indexes 4 and 6 were considered positive (2+) as the staining index 9 (3+).

Samples of feline mammary carcinomas with previous known ER/PR/HER2 status were used as controls, whereas a feline tonsil tissue sample was used as a positive control for the assessment of Ki-67 index and CXCR4, according to the manufacturer's instructions. Tissue sections without incubation of primary antibodies were used as negative controls.

All slides were independently subjected to blind scoring by two independent pathologists and discordant interpretations were further debated and settled using a multiobserver microscope. Images were taken with a color optical microscope system (Axiovert S100 with AxioCam HRc; Carl Zeiss BV, Sliedrecht, the Netherlands) and analyzed using AxioVision (Carl Zeiss).

### Quantification of serum SDF-1 and HER2 levels by ELISA

Considering the extensive sequence homology between the human SDF-1 ligand and human HER2 receptor with theirs homologues in Felis catus (96% and 93%, respectively), serum SDF-1 and HER2 levels were evaluated by using two commercial ELISA-based kits suitable to use in humans (CXCL12/SDF-1 DuoSet ELISA kit, R&D Systems, Minneapolis, USA; sHER2 Platinum ELISA kit, eBioscience, San Diego, USA), following the manufacturer's protocol. Briefly, for each ELISA assay, a standard curve was generated using seven dilutions of the recombinant SDF-1 or HER2 protein, with known concentrations. Then, the first row of a 96-well ELISA plate was coated with 100 μl/well of each rSDF-1 or rHER2-ECD dilution, in duplicate, on “standards wells”, whereas 10 μl of each serum sample was added to 90 μl of assay buffer in “sample wells”, also in duplicates. After two consecutive washes (2×300μl with Wash Buffer), 50 μl of an HRP-conjugated mouse anti-IgG was added to each well and incubated at 37°C, for 2 hours, on a microplate shaker at 100 rpm. After a second washing step (3×300 μl), 100 μl of the 3,3′,5,5′-tetramethyl-benzidine (TMB) substrate solution was added to each well and the final mixture was incubated at RT, for 10 min, in the dark.

For quantification of serum SDF-1 levels, a 96-well ELISA plate was coated overnight with 1 μg/ml of mouse anti-human SDF-1 capture antibody (100 μl) in 1% bovine serum albumin (BSA) - phosphate buffer solution (PBS). After several washes (0.05% Tween-20 in PBS), each well was blocked (1% BSA PBS) for 1h to prevent non-specific binding and 100 μl of diluted serum samples (1:10) and standards were incubated for 2h. The plate was washed and 50ng/ml of the biotinylated goat anti-human SDF-1 detection antibody (100 μl) was added to each well for 1h incubation. Conjugated streptavidin-horseradish peroxidase (HRP) was diluted 40 times and incubated in the plate wells for 45 min after previous washes. A final wash was performed before adding 100ul of the HRP substrate (3,3′,5,5′-tetramethylbenzidine) solution (R&D Systems, Minneapolis, USA). After 25 min of incubation in the dark, the reaction was stopped with 50 μl of 2N sulfuric acid and the absorbance was measured in a spectrophotometer (LabSystems IEMS Reader MF, Labsystems/Thermo Scientific, Helsinki, Finland) using 450 nm as the primary wavelength and 570 nm as reference wavelength. Quantification of serum HER2 levels was performed as previously reported by us [7].

### Statistical analysis

Graphpad Prism version 7.02 (La Jolla, USA) was used for all statistical analysis and a two-tailed *p* value less than 0.05 was considered statistically significant. Outliers were removed from analysis based on the combination of **Ro**bust regression and **Out**lier removal, ROUT method [65] implemented in Graphpad Prism software. This method identifies outliers from nonlinear curve fits with reasonable power and few false positives [65]. The non-parametric Mann-Whitney test was used to compare the serum SDF-1 levels between healthy cats and cats with mammary carcinoma, and cats with CXCR4-negative and CXCR4-positive mammary carcinomas. Receiver-operating characteristics (ROC) curves were performed to choose the best cut-off value for serum SDF-1 levels using ELISA and to determine the sensitivity and specificity of the assay. The non-parametric Kruskall-Wallis test and the Dunn's multiple comparisons post-test were used to compare serum SDF-1 levels in healthy cats and cats with different mammary carcinoma subtypes. The Fisher's exact test was used to assess the associations between serum SDF-1 levels and clinicopathological features (categorical variables, placed in ordinal or nominal scale). The correlation coeficient of Pearson was calculated to correlate the serum SDF-1 levels with the clinicopathological features measured in a metric scale (continuous variables).

OS and DFS were analyzed by the Kaplan–Meier method (log-rank test). Overall survival (OS) period was defined as the time elapsed between the initial diagnosis and the death/euthanasia due to tumor metastasis. Disease-free survival (DFS) time was calculated from the date of surgery to the date of relapse (local, in other mammary gland or in distant organs) or death from cancer-related causes. Survival curves were estimated using the Kaplan-Meier method and the Log-rank test to compare the outcome (OS median and DFS median), regarding serum SDF-1 levels. Finally, animals that died from a disease unrelated to mammary tumors or were lost during the follow-up were excluded for the OS analysis.

## Abbreviations

AUC: area under the curve
BSA: bovine serum albumin
CI: confidence interval
CXCL12: chemokine ligand 12
CXCR4: C-X-C Chemokine Receptor Type 4
CXCR7: C-X-C Chemokine Receptor Type 7
DFS: disease free-survival
ELISA: enzyme-linked immunosorbent assay
ER: estrogen receptor
ERK1/2: extracellular signal-regulated kinase 1/2
FMC: feline mammary carcinoma
GPCR: chemokine receptors G protein-coupled receptors
HER2: human epidermal growth factor receptor type II
HIF-1α: hypoxia inducible factor 1 alpha
HRP: horseradish peroxidase
IFN-γ: interferon-gamma
LA: Luminal A
LB: Luminal B
MAPK: mitogen-activated protein kinase
NF-κB: nuclear factor kappa light-chain-enhancer of activated B cells
OR: odd ratio
OS: overall survival
PBS: phosphate buffer solution
PI3K: phosphoinositide 3-kinase
PR: progesterone receptor
*r*: Spearman correlation coefficient
ROC: receiver-operating characteristics
SDF-1: stromal cell-derived-factor-1
SEM: standard error of mean
sHER2: serum HER2
TBS: tris-buffered saline
TGF-β1: transforming growth factor-beta 1
TN: triple negative
VEGF: vascular endothelial growth factor
